# Green Tea Intake Reduces High-Fat Diet-Induced Sensory Neuropathy in Mice by Upregulating the Antioxidant Defense System in the Spinal Cord

**DOI:** 10.3390/antiox14040452

**Published:** 2025-04-10

**Authors:** Gessica Sabrina de Assis Silva, Thalita da Cruz Monteiro Santana, Ana Carolina Lucchese Velozo, Ana Paula Azevêdo Macêdo, Mariane dos Santos Gonçalves, Ricardo David Couto, Milena Botelho Pereira Soares, Max Denisson Maurício Viana, Cristiane Flora Villarreal

**Affiliations:** 1 School of Pharmacy, Federal University of Bahia, Salvador 40170290, BA, Brazil; gsabrinaassis@gmail.com (G.S.d.A.S.); thalitacms@ufba.br (T.d.C.M.S.); anavelozo@ufba.br (A.C.L.V.); paulamacedo.nut@gmail.com (A.P.A.M.); marianesg1@gmail.com (M.d.S.G.); rdc@ufba.br (R.D.C.); max.viana@ufba.br (M.D.M.V.); 2 Gonçalo Moniz Institute, Oswaldo Cruz Foundation (FIOCRUZ), Salvador 40296710, BA, Brazil; milena.soares@fiocruz.br; 3 Institute of Advanced Systems in Health (ISI-SAS), Senai Cimatec, Salvador 41650010, BA, Brazil

**Keywords:** green tea, antioxidant, neuropathy, high-fat diet, oxidative stress

## Abstract

One of the most common complications of obesity is peripheral nerve damage, which progresses to sensory neuropathy. Green tea (GT) intake has been associated with weight loss and metabolic biomarkers modulation due to its antioxidant properties. The present work characterized the effects of GT in high-fat diet (HFD)-induced neuropathy and investigated the mechanisms involved. C57BL/6J male mice were fed an HFD or control diet, associated with GT or vehicle intake for 16 weeks. Weight, blood glucose, and nociceptive thresholds were assessed. Morphological and morphometric analyses of the sciatic nerves were performed. Activation of the cellular antioxidant system in the spinal cord was assessed by real-time PCR. GT intake reduced weight gain, hyperglycemia, and the development of sensory neuropathy. Furthermore, in HFD-fed mice that consumed GT, the morphology of the sciatic nerve was preserved. RT-qPCR analysis showed that HFD-fed mice ingesting GT had higher spinal levels of superoxide dismutase, catalase, glutathione peroxidase, and nuclear factor erythroid 2-related factor 2 (NRF2) mRNA compared to the HFD-fed mice ingesting vehicle, suggesting that the endogenous antioxidant system was more activated in response to GT consumption. In conclusion, the data suggest that GT intake reduces HFD-induced neuropathy, probably by upregulating antioxidant gene expression.

## 1. Introduction

Peripheral neuropathy (PN) is a common and debilitating complication of metabolic conditions such as metabolic syndrome, obesity, prediabetes, and type 2 diabetes. PN typically manifests as sensory loss, paresthesia, dysesthesia, neuropathic pain, and gait disturbance, representing a frequent cause of disability and reduced quality of life [1]. These sensory alterations that characterize PN reflect changes in the functional pattern of nociceptive neurons, which result from damage to peripheral nerves, with changes in myelin fibers and axonal degeneration [2].

Although the pathogenesis of PN is not fully understood, studies have pointed to weight gain, sustained hyperglycemia, and redox imbalance as drivers of this condition [3,4]. In fact, high-fat diets (HFDs) and hyperglycemia have been identified as important risk factors for the development of PN due to neuronal damage resulting from increased oxidative stress [4,5,6,7]. HFDs increase reactive oxygen species [8] and induce overexpression of pro-inflammatory mediators [9] involved in neuronal inflammation associated with the development of nociceptive hypersensitivity and nerve fiber changes observed in HFD-fed mice [10]. Therefore, murine models of HFDs develop features of prediabetes, obesity, and PN, as seen in humans, making them a useful tool for the development of novel targeted therapeutic agents [11,12].

The pharmacological management of PN is challenging, as many patients respond poorly to current treatments [13]. Many clinical practice guidelines have been published in recent years to guide the choice of the most appropriate drugs for the control of neuropathic pain; however, it is often resistant to over-the-counter analgesics [14]. To overcome the limitations of pharmacotherapy, different approaches of treatments have been explored, aiming to develop new therapeutic strategies to control sensory deficits and refractory pain.

Green tea (GT) is a widely popular beverage, and its regular intake has been reported to have several health benefits [15]. Its secondary metabolites include catechin, amino acids, polyphenols, caffeine, and others, compounds related to multiple pharmacological properties, such as hypolipidemic, anti-inflammatory, and antioxidant activities [16,17]. Green tea catechins inhibit the activation of glial cells, consequently reducing neuroinflammation in the nervous system [18], which is a key event involved in neurological diseases, including neuropathic pain. Indeed, under experimental conditions, GT intake reduces sensory alterations and pain-like behavior in peripheral neuropathy induced by oxaliplatin or by nerve constriction [19,20].

Considering these findings, associated with the demonstration that GT improves systemic metabolism and decreases body weight [21], the present study was designed to evaluate the influence of daily GT intake in a model of HFD-induced sensory neuropathy. To better understand the mechanisms involved, the influence of GT intake on the activation of the endogenous antioxidant system and on the morphological and morphometric parameters of the peripheral nerve was evaluated.

## 2. Materials and Methods

### 2.1. Preparation of Green Tea

GT was purchased from Viva Natureza^®^ Co., Ltd. (São Gonçalo dos Campos, BA, Brazil). GT was prepared by infusing 20 g of tea leaves in tea bags soaked in 1 L of boiled water (equivalent to 1 tea bag per 200 mL of water to simulate an average tea infusion) in a covered container for 3 min. The tea bags were then squeezed out of excess water and GT was placed in the animal bottles. Preparations were made daily. The chemical composition of the GT used in the present work was characterized by high performance liquid chromatography (HPLC), and the results were described in a previously published work [22].

### 2.2. Animals

Experiments were performed on adult male C57BL/6J mice (24–28 g) obtained from the Animal Facilities of the Gonçalo Moniz Institute, FIOCRUZ (Salvador, BA, Brazil). Inclusion criteria were sex (male mice) and body weight (24–28 g). Female mice were not used in this study because of the influence of hormonal fluctuations throughout the estrous cycle on the perception of painful stimuli. Mice were housed in micro-isolator cages with polycarbonate igloos as environmental enrichment, in a temperature-controlled room (22–24 °C), under a 12:12 h light/dark cycle of artificial light, with monitored access to water and food. Groups of mice were formed by cluster random sampling, with two cages housing three mice each being randomly selected and assigned to a group (n = 9). Group size was determined based on previous studies that employed the same model [23]. No mice from the experimental groups were excluded from the analysis. All behavioral tests were performed between 8:00 a.m. and 5:00 p.m. by researchers working in pairs. While one of them was responsible for handling mice and administering drugs, behavioral data were acquired by a blind evaluator. If a mouse displayed signs of extreme pain or distress or any noticeable behavioral alteration it would be discontinued from the experiment and euthanized—during the entire study, there was no need to force this endpoint. Animal care and handling procedures were in strict accordance with the recommendations of the Code of Practice for the Housing and Care of Animals Used in Scientific Procedures [24] and the research was designed according to the Three Rs concept [25]. All protocols were reviewed and approved by the Institutional Animal Care and Use Committee of the Federal University of Bahia (CEUA/EMEVZ-UFBA, number: 03/2019; 24 April 2019). Efforts were made to minimize animal distress. Behavioral tests were performed by blind evaluators.

### 2.3. High-Fat Diet (HFD)-Induced Neuropathy Model

The high-fat diet-induced neuropathy model was performed in C57BL/6J mice as previously described by Pereira et al. [23] with slight adaptations. Briefly, mice were fed a high-fat diet or a control diet for 16 weeks. The HFD (Pragsoluções Biociências^®^, Jaú, SP, Brazil) consisted of 17.5% protein, 57.9% fat, and 24.7% carbohydrate with adequate levels of minerals and vitamins. The control diet (CD; Pragsoluções Biociências^®^, Jaú, SP, Brazil) consisted of 16.4% protein, 10.5% fat, and 73.1% carbohydrate with adequate levels of minerals and vitamins (Table S1). Mice on both diets also ingested vehicle (water) or green tea during the experimental period. Measurements of body weight and blood glucose were performed throughout the experimental time. Body weight was measured using a precision digital scale (Ohaus Adventurer^®^ AR2140, Parsippany, NJ, USA). Blood glucose was determined in tail vein blood samples using glucose sticks (Accu-Check^®^, Itapevi, SP, Brazil). Pain-like behavior was assessed by the von Frey test throughout the experimental period to confirm the development of PN.

### 2.4. Experimental Design

A total of 36 mice were randomized into four groups (n = 9): control diet plus vehicle (CD); control diet plus green tea (CD + GT); high-fat diet plus vehicle (HFD); high-fat diet plus green tea (HFD + GT). Tea beverages were the sole source of drinking fluids for the GT groups. All drinking fluids were bottled and replaced daily with fresh water or GT. Diets and beverages were consumed for 16 weeks. Water and GT consumption were checked daily using a beaker, resulting from the difference between the solution initially placed and the remaining volume. Liquid consumption measurements were always made at the same time. Food consumption was measured from the first to the last day as described by Pereira et al. [23]. Every two days, 100 g of the control diet or high-fat diet were placed in each polypropylene box, and the individual daily consumption of the animals was obtained by averaging the consumption, subtracting the remaining feed of two days, divided by the number of animals per box. Weight assessments were performed at baseline and every 2 weeks, and blood glucose was measured at baseline and every 4 weeks throughout the experimental period. In order to demonstrate the development of neuropathy and the impacts of GT intake, the nociceptive threshold was measured with von Frey filaments at baseline and once a week after the introduction of HFD. At the end of the experimental period (16 weeks after the start of HFD and treatments), mice were euthanized by an overdose of intraperitoneal sodium thiopental (50 mg/kg; Cristália, Itapira, SP, Brazil) followed by cervical dislocation to ensure death. Sciatic nerves and spinal cord (L4–L5) samples were then obtained for morphological and morphometric analyses (n = 3) and for gene expression assessment (n = 6) by quantitative real-time polymerase chain reaction (qRT-PCR).

### 2.5. Von Frey Test for Assessment of Mechanical Nociceptive Threshold

The pain-like behavior that characterizes PN was assessed throughout the experimental period using the von Frey test, which measures the mechanical nociceptive threshold. Von Frey filaments consist of nylon thread segments with logarithmically incremental stiffness (0.008–0.6 g) previously determined by the manufacturer (Stoelting^®^, Chicago, IL, USA). Mice were placed in transparent acrylic boxes on a wired grid floor. After an adaptation period of 20 min, the right hind paw was touched vertically with a series of filaments with varying stiffness until they were slightly bent. The sudden withdrawal of the touched paw was considered a positive response. Mechanical nociceptive thresholds were determined by the up-and-down method, as previously described [26]. The development of PN was characterized by mechanical allodynia, indicated by the reduction in the paw withdrawal threshold (in grams).

### 2.6. Morphological and Morphometric Analyses of Sciatic Nerve

Sciatic nerve samples (±1 cm) were processed and subjected to morphological and morphometric analysis by light microscopy. Samples were fixed in 2.5% glutaraldehyde (grade I, Sigma-Aldrich, St. Louis, MO, USA) in 0.1 M sodium cacodylate buffer (Sigma-Aldrich) overnight; washed in cacodylate buffer (Sigma-Aldrich); post-fixed in 1% osmium tetroxide (Sigma-Aldrich), 0.8% potassium ferricyanide (Êxodo Científica, Sumaré, SP, Brazil), and 5 mM CaCl_2_ (Dinâmica^®^, Indaiatuba, SP, Brazil) in the same buffer for 60 min; serially dehydrated using graded acetone (Dinâmica^®^); and embedded in Poly/Bed resin (Polysciences, Warrington, PA, USA). Cross sections (1 μm thick) were stained with 1% toluidine blue and examined by light microscopy. Semi-thin section images were captured and examined using Image-Pro Plus 7.01 software (MediaCybernetics, Rockville, MD, USA). Morphometric parameters of myelinated fibers, such as axonal diameter, fiber diameter, and myelin sheath thickness, were obtained as previously described [27,28,29].

### 2.7. Real-Time PCR

The transcription of superoxide dismutase (*Sod*), catalase (*Cat*), glutathione peroxidase (*Gpx*), and nuclear factor erythroid 2-related factor 2 (*Nrf2*) genes was evaluated by qRT-PCR in mouse spinal cord at the conclusion of the experimental period (16 weeks after the onset of the diet and GT consumption), as previously described [30]. Total RNA was extracted from L4–L5 spinal segments with Trizol reagent (Invitrogen, Carlsbad, CA, USA), and the concentration was determined by photometric measurement. A High-Capacity cDNA Reverse Transcription Kit (Applied Biosystems, Foster City, CA, USA) was used to synthesize cDNA from 1 μg of RNA, according to the manufacturer’s recommendations. Synthesis of cDNA and RNA expression analysis was performed by qRT-PCR using TaqMan Gene Expression Assay (Thermo Fisher Scientific, Waltham, MA, USA) for *Sod1* (Mm01344233_g1), *Cat* (Mm00437992_m1), *Gpx1* (Mm00492427_m1), and *Nrf2* (Mm00477784_m1). A no-template control (NTC) and no-reverse transcription controls (No–RT) were also included. All reactions were run in triplicate on an ABI7500 Sequence Detection System (Applied Biosystems) under standard thermal cycling conditions. The mean Ct (cycle threshold) values from triplicate measurements were used to calculate the expression of the target gene, with normalization to an internal control *Gapdh* (Mm99999915_g1).

### 2.8. Statistical Analysis

Data are presented as means ± standard deviation (SD) of measurements made on 9 animals in each group. Comparisons between three or more groups were made using one-way ANOVA with Tukey’s post hoc test (analyses of morphological and morphometric parameters of sciatic nerve fibers and gene expression in the spinal cord) or, for repeated measures (body weight, blood glucose, and mechanical nociceptive threshold data), two-way ANOVA with Bonferroni’s post hoc test. All data were analyzed using Prism 8 Computer Software (GraphPad, San Diego, CA, USA). Statistical differences were considered significant at *p* < 0.05.

## 3. Results

### 3.1. GT Prevents Weight Gain and Increased Blood Glucose Levels Caused by High-Fat Diet 

**Figure 1:**
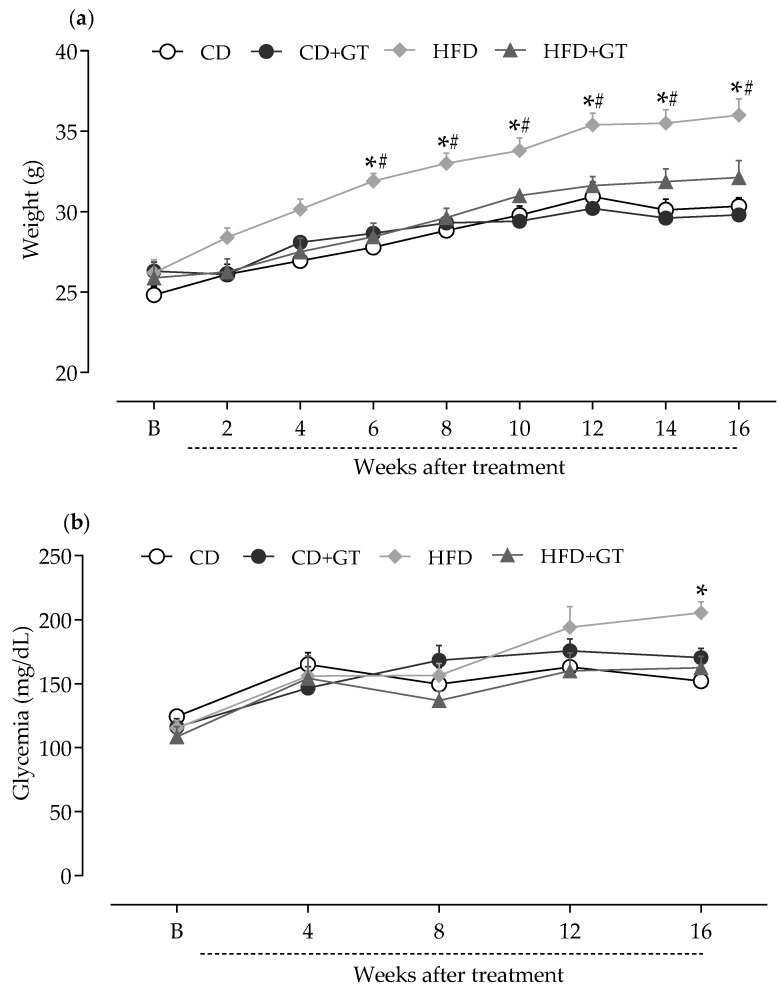
Effects of green tea intake on body weight and blood glucose levels in mice fed a high-fat diet. Mice were fed a control diet receiving vehicle (CD) or green tea (CD + GT), or a high-fat diet receiving vehicle (HFD) or green tea (HFD + GT) for 16 weeks: (**a**) Body weight and (**b**) blood glucose (ordinate axis) were measured before (baseline, B) and after the start of beverage intake at different times throughout the experimental period (abscissa axis). Data are expressed as means ± SD; n = 9 mice per group. * *p* < 0.05 compared to CD group; ^#^ *p* < 0.05 compared to HFD + GT group, as determined by two-way ANOVA followed by Bonferroni’s test.

The impact of the high-fat diet and the effects of chronic green tea intake on body weight and blood glucose levels in mice were initially evaluated (Figure 1). Mice were fed HFD or CD and ingested GT or vehicle (water) for 16 weeks. Mice from different experimental groups (n = 9) showed similar body weight in baseline measurements (B; Figure 1a). As illustrated in Figure 1a, HFD-fed mice gained significantly more body weight compared to CD-fed groups (*p* < 0.05). GT intake significantly prevented body weight gain in HFD-fed mice throughout the experimental period (*p* < 0.05), compared to the vehicle-treated HFD group. There were no significant differences in body weight between groups ingesting GT under different diets at any time point.

The influence of GT intake on the blood glucose levels of mice was also monitored throughout the experimental period (Figure 1b). Animals started in similar glycemic conditions (B, baseline; Figure 1b). HFD caused an increase in blood glucose levels in mice at the 16th week compared to the CD-fed group (*p* < 0.05). GT intervention prevented the glucose increase in mice caused by HFD. These findings corroborate the metabolic changes induced by the HFD diet in the experimental model as well as evidence of the beneficial effects of GT consumption on glycemic control and body weight.

### 3.2. GT Intake Reduces the Development of Neuropathy in High-Fat Diet -Fed Mice

**Figure 2:**
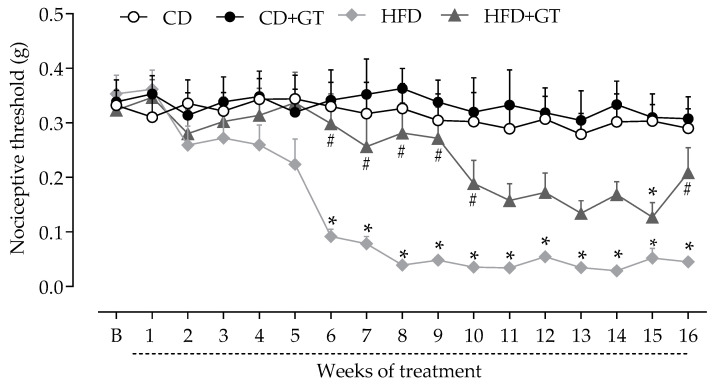
Antinociceptive effects of green tea intake on high-fat diet-induced sensory neuropathy. Mice were fed a control diet receiving vehicle (CD) or green tea (CD + GT), or a high-fat diet receiving vehicle (HFD) or green tea (HFD + GT) for 16 weeks. Mechanical nociceptive thresholds (ordinate axis) represent the filament weight (g) to which mice respond in 50% of trials and were assessed before (baseline; B), and after the start of beverage intake at different times throughout the experimental period (abscissa axis). Data are expressed as means ± SD; n = 9 mice per group. * *p* < 0.05 compared to CD group; ^#^ *p* < 0.05 compared to HFD group, as determined by two-way ANOVA followed by Bonferroni’s test.

Behavioral tests were performed at baseline and weekly throughout the experimental period, to evaluate the effects of GT intake on the sensorial parameter of HFD-induced neuropathy. Mice from different experimental groups (n = 9) showed similar mechanical thresholds in baseline measurements (B, Figure 2). The development of sensory neuropathy was characterized by a reduction in the mechanical nociceptive threshold in the HFD group compared to the CD group (*p* < 0.05), starting from the 6th week and remaining throughout the experimental period. GT consumption reduced the development of neuropathy associated with HFD intake, as nociceptive thresholds in the HFD + GT group were statistically different from the CD group only at the 15th-week time point (*p* < 0.05).

### 3.3. GT Intake Preserves Morphology of Myelinated Sciatic Nerve Fibers in High-Fat Diet-Induced Neuropathy

**Figure 3:**
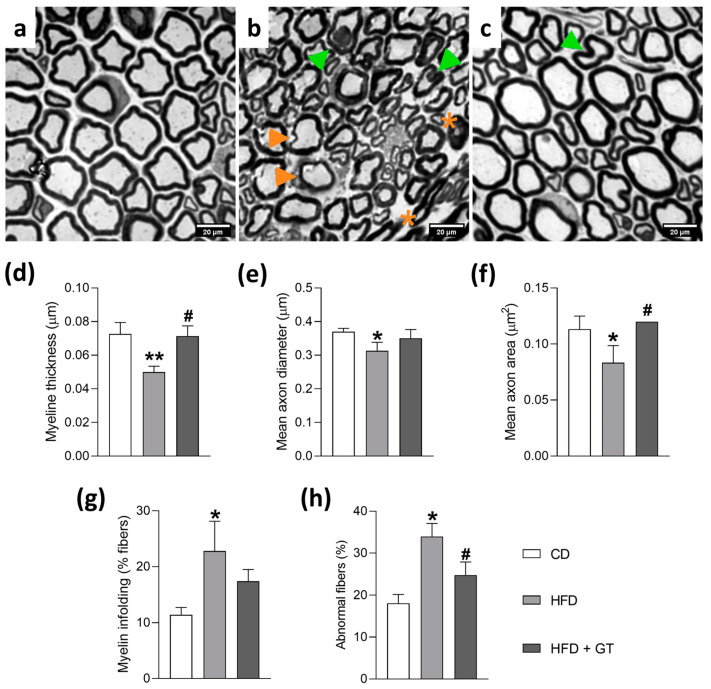
Effects of green tea intake on morphological and morphometric parameters of myelinated fibers of the sciatic nerve of mice in a model of neuropathy induced by a high-fat diet. Panels (**a**–**c**): photomicrographs of sciatic nerves from mice fed a control diet (CD; (**a**)), high-fat diet + vehicle (HFD; (**b**)), or high-fat diet + green tea (HFD + GT; (**c**)) for 16 weeks. Light microscopy analysis revealed that the sciatic nerve of HFD-fed mice (**d**–**h**) had thinned myelinated fibers with loose (orange arrowheads) and thinner myelin sheath, decreased axonal area (orange asterisks), and abnormal myelin with infoldings into the axoplasm (green arrowheads). Panel (**c**) shows that the sciatic nerve of HFD-fed and GT-treated mice had myelin fibers with normal morphology. Scale bar = 50 μm. Data are expressed as means ± SD; n = 3 mice per group. * *p* < 0.05, ** *p* < 0.01 compared to CD group; ^#^ *p* < 0.05 compared to HFD group, as determined by one-way ANOVA followed by Tukey’s test.

It is well established that the sensory changes associated with peripheral neuropathies reflect damage to peripheral nervous tissue, including changes in myelin fibers and axonal degeneration of nerve fibers [1,2]. Thus, the effects of GT consumption on morphological and morphometric parameters of the sciatic nerve’s myelinated nerve fibers of HFD-fed mice were investigated at the end of the experimental period (16 weeks). Figure 3 shows photomicrographs of the sciatic nerve of mice representative of each experimental group (n = 3). Figure 3a shows the sciatic nerve of mice from the CD group, presenting myelinated fibers of variable calibers and regular contours, intact myelin sheath, and sheath thickness proportional to the diameter of the respective axons. A light microscopy image revealed that the sciatic nerve of HFD-fed mice displayed thinned myelinated fibers loss and thinner myelin sheath, decreased axonal area, and increased irregularly shaped fibers and myelin infoldings (Figure 3b). Furthermore, quantitative morphometric analyses showed that all observed parameters were altered in the HFD-fed group compared to the CD group (Figure 3d–h, *p* < 0.05 for all parameters analyzed), indicating axonal degeneration. On the other hand, in mice fed with HFD and treated with GT, the sciatic nerve presented myelin fibers of variable calibers with preserved normal morphology (Figure 3c), compared to the HFD group (Figure 3b). Mice from the HFD + GT group presented morphometric parameters similar to those of the CD group (Figure 3d–h). The evaluated parameters of myelin sheath thickness, axonal diameter, axonal area, myelin fiber shape, and myelin infoldings were not different between the CD and HFD + GT groups, indicating that GT consumption prevented the morphological changes induced by HFD.

### 3.4. GT Intake Induces Upregulation of Endogenous Antioxidant System Components in the Spinal Cord of Mice in the High-Fat Diet -Induced Neuropathy Model

**Figure 4:**
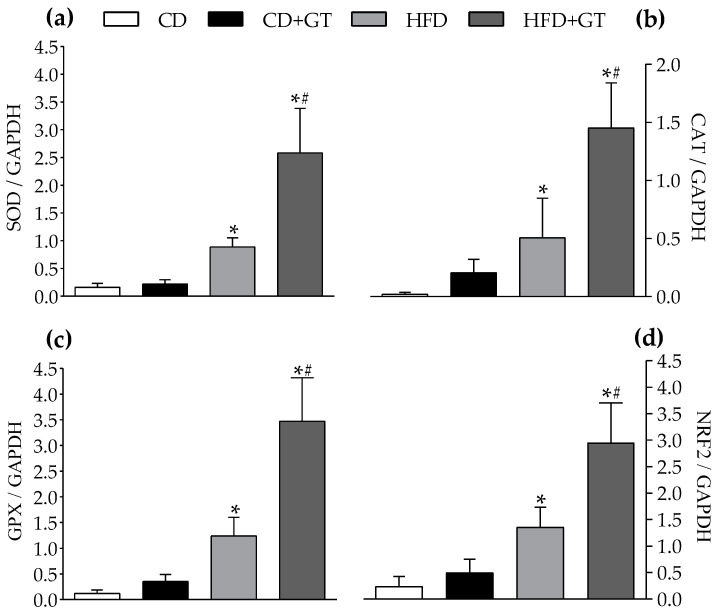
Effects of green tea intake on antioxidant gene expression in the spinal cord of mice during high-fat diet-induced neuropathy. Mice were fed a control diet receiving vehicle (CD) or green tea (CD + GT), or a high-fat diet receiving vehicle (HFD) or green tea (HFD + GT) for 16 weeks. Spinal mRNA levels were measured by RT-qPCR at the end of the experimental period. The y-axis shows the mRNA expression of the target gene normalized by the constitutive *Gapdh* gene. Panels show the spinal levels of superoxide dismutase mRNA (SOD; (**a**)), catalase mRNA (CAT; (**b**)), glutathione peroxidase mRNA (GPX; (**c**)), and nuclear factor erythroid 2-related factor 2 mRNA (NRF-2; (**d**)) of mice in the last daily treatment. Data are expressed as means ± SD; n = 6. * *p* < 0.05 compared to CD group; ^#^ *p* < 0.05 compared to HFD group, as determined by one-way ANOVA followed by Tukey’s test.

Considering the well-established antioxidant properties of GT, as well as the key role of oxidative stress in pain pathways for the induction and maintenance of sensory neuropathy described in the literature [4,26,31], an experimental protocol was designed to investigate whether GT is able to modulate antioxidant pathways in the spinal cord of mice (n = 9). Data obtained by RT-qPCR assay showed that HFD-fed mice had increased levels of superoxide dismutase (Figure 4a), catalase (Figure 4b), glutathione peroxidase (Figure 4c), and NRF2 (Figure 4d) mRNA in the spinal cord compared to CD-fed mice (*p* < 0.05). In the HFD-GT group, significantly higher mRNA levels of these antioxidant factors were observed in the spinal cord compared to the HFD group (*p* < 0.05). These results suggest that spinal expression of antioxidant genes is upregulated in the HFD-induced neuropathy model, and that GT consumption enhances this protective response.

## 4. Discussion

The therapeutic potential of GT intake in weight control [17,21], improvement of metabolic markers [15,17,21], and control of pain [19,20] has been evidenced. However, its effect on HFD-induced sensory neuropathy has not yet been investigated. In the current study, HFD-fed mice that consumed GT showed a significant reduction in the development of sensory neuropathy. This effect was accompanied by weight and blood glucose control, and preservation of normal morphology of myelinated sciatic nerve fibers. These effects are probably associated with the antioxidant properties of GT, considering that GT consumption increased the production of antioxidant defenses in the nervous tissue. Considering the involvement of metabolic disorders and oxidative stress in the pathophysiology of PN, the data presented here highlight the therapeutic potential of GT consumption for the control of HFD-induced PN.

First, the effects of GT consumption on the body weight of HFD-fed mice were evaluated. Statistical differences were observed between the control and HFD groups, the HFD group showed a gradual gain in body weight, which can be attributed to the higher caloric values of the HFD [32]. GT consumption completely prevented weight gain in HFD-fed mice throughout the entire experimental period. These results are in line with previous reports that GT intake is able to control appetite and body weight gain in mice. Sayama et al. [33] demonstrated that the gradually increased incorporation of GT (1–4%) in the diet can reduce mice’s appetite and weight gain. These effects have also been described in clinical trials and validated by meta-analysis studies [34,35]. GT catechins mixed with caffeine have been proposed in body-weight management, especially by sustained energy expenditure, fat oxidation, ghrelin secretion inhibition, and preservation of fat-free body mass [36].

HFDs are known to increase glucose levels in mice. So, the influence of GT intake on the glycemia of mice was monitored throughout the experimental period. GT consumption prevented the glucose increase caused by HFD in mice. Corroborating the results presented, Snoussi et al. [37] reported that HFD-fed rats treated with GT showed improvement in glucose tolerance and control of glycemic homeostasis by reducing sodium-glucose transport proteins 1 (SGLT-1) and increasing glucose transporter 2 (GLUT2) levels in the jejunal mucosa. In this same study, the administration of synthetic phenolic compounds present in GT, especially epicatechin, selectively inhibited SGLT-1 activity, suggesting the importance of this target for the glycemic control mechanism mediated by GT.

SGLT-1 is a transporter located in the apical membrane of intestinal epithelial cells, which acts in the transport of glucose during fasting [38]. SGLT-1 is also considered a key molecule in sensing glucose flux and is highly regulated by peptides and hormones, such as peptide YY (PYY) [39]. PYY is a gut hormone crucial in regulating food intake, which can suppress appetite and control blood glucose levels. Ma et al. [40] showed increased stimulation of PYY secretion by GT intake in a model of HFD-induced obesity. Thus, collectively, these findings may explain the effects of GT consumption on body weight and glycemia control in HFD-fed mice observed in the present study.

It has been well documented in the literature that obesity and hyperglycemia induce metabolic disorders that generate oxidative stress and damage to peripheral nerve fibers, contributing to the development of sensory neuropathy [3,4]. In line with this concept, in the present work, the effects of GT intake on the nociceptive threshold were investigated in HFD-fed mice. Mechanical allodynia, characterized by a reduction in the mechanical nociceptive threshold, was observed in mice from the 6th week after the introduction of HFD and remained throughout the 16-week experimental period. Mechanical allodynia is a sensory alteration observed in HFD-induced neuropathy in mice [11,41], and is also manifested in humans with painful PN [3]. Interestingly, in our study, the mechanical allodynia indicative of the development of sensory neuropathy began in parallel with the increase in body weight in HFD-fed mice. The finding that daily administration of GT reduced the behavioral signs of sensory neuropathy is in agreement with other studies. Lee et al. [19], in an experimental study of oxaliplatin-induced PN in rats, observed that oral administration of GT alleviated sensory symptoms, such as allodynia, after 6 weeks of treatment. Intraperitoneal administration of GT has also been shown to alleviate mechanical allodynia in a mouse model of sensory neuropathy induced by chronic nerve constriction [20]. In common with both studies, the antinociceptive effect induced by GT was related to the therapeutic properties of its secondary metabolites. In fact, methylxanthines, polyphenolic catechins, epicatechin gallate, and epigallocatechin gallate, present as major components in the GT sample of the present work, have been reported to promote antinociception in models of pain [42,43,44].

It has also been described that PN can manifest as damage to nervous tissues, markedly with loss of myelin and distal axonal degeneration of sensory nerve fibers [2]. These structural changes in sensory fibers, and the consequent modifications in their functional pattern, are reflected in the sensory changes that characterize PN, including pain and allodynia. Considering that GT consumption was able to reduce the development of HFD-induced allodynia, the hypothesis that GT may prevent damage to peripheral nerve sensory fibers was next investigated. Thus, at the end of the experimental period (16th week), samples of the sciatic nerve were collected and different morphometric parameters were analyzed. Morphometry of the sciatic nerve showed significant differences between myelinated fibers of HFD-fed mice and control mice, such as the presence of thinned myelinated fibers, loose myelin sheath, and decreased axonal area. The present data are consistent with the study of Ozay et al. [4], which showed that HFD can exert damage to the sensory fibers of the peripheral nerves. It has been proposed that alterations in genes and signaling pathways involving oxidative stress of lipid metabolism, and neuroinflammation may contribute to neuronal damage in the peripheral nervous system of HFD-fed mice [5,45].

Importantly, GT was able to prevent the morphologic alterations in HFD-fed mice, considered to be hallmarks of PN, indicating that daily intake of GT prevented the evolution of PN. In line with these findings, Chen et al. [46] determined that GT polyphenols can promote functional recovery and nerve regeneration in rats after peripheral nerve injury. The authors attributed the effects to increased expression of mediators involved in neuron survival and differentiation and myelin formation and maintenance. Although the mechanisms by which GT reduces morphological alterations of sensory fibers of peripheral nerves during neuropathies are not fully comprehended, the involvement of GT polyphenols has been proposed. Polyphenolic compounds are GT bioactives with relevant modulatory properties on oxidative stress and inflammation mediators, making these secondary metabolites capable of protecting nervous tissue and promoting its functional recovery, attenuating sensory neuropathy in rodents [46,47,48].

In fact, neuronal damage due to oxidative stress has been considered a central pathophysiological mechanism of HFD-induced PN [4]. To validate the hypothesis that GT intake alleviates HFD-induced neuropathy by modulating the antioxidant system, the expression of genes that constitute endogenous antioxidant defenses was assessed by RT-qPCR. The data consistently demonstrated that HFD-induced sensory neuropathy is associated with enhanced spinal mRNA levels of superoxide dismutase (SOD), catalase (CAT), glutathione peroxidase (GPX), and nuclear factor erythroid 2-related factor 2 (NRF2). These data may suggest the presence of oxidative stress in the spinal cord microenvironment of HFD-fed mice, although the present study did not investigate this phenomenon. On the other hand, the study by Ozay et al. [4] corroborates this hypothesis, as it showed that elevated levels of oxidative stress markers occur in parallel with increased activity of antioxidant enzymes in nervous tissue in the model of neuropathy induced by a high-fat diet.

Oxidative stress-induced damage is a hallmark of sensory neuropathies and has been implicated in the pathophysiological changes underlying several disease-related complications [31]. Specifically in sensory neuropathy caused by HFD, oxidative stress damage has been reported in areas of the somatosensory system involved in the transmission of nociceptive input, particularly in the spinal cord. An imbalance between the generation of reactive oxygen species, and in the levels of antioxidant defense, results in oxidative stress and neuroinflammation, involved in the maintenance of neuropathic pain [31,45].

Importantly, in the present study, the gene expression analysis revealed that the endogenous antioxidant system was upregulated in the spinal cord by GT consumption in HFD-induced neuropathic mice. These effects may be related to the synergism of secondary metabolites present in this sample of GT, such as caffeine (34.4%), epicatechin gallate (11.9%), and epigallocatechin (5.8%), which have well-described antioxidant properties [22]. Corroborating the present results, Jing et al. [47] demonstrated that GT catechin attenuates neuropathic pain induced by nerve constriction in rats through the triggering of the NRF2-induced antioxidant system. According to present data, it is possible to propose that GT intake can attenuate HFD-induced sensory neuropathy by enhancing the antioxidant defense system in the spinal cord of mice. On the other hand, the decrease in blood glucose and body weight induced by GT may also contribute to the reduction in neuropathy progression in GT-treated mice, although it is not possible to conclude the relevance of each of these mechanisms for the effects of GT on HFD-induced neuropathy.

Although no effective treatment for HFD-induced PN has been found, many prevention and treatment strategies have been suggested, including those with natural antioxidants [33,37,40]. Therefore, the present study provides scientific data that reinforce the potential of GT to prevent the progression of peripheral neuropathy, although clinical studies are still needed to confirm this therapeutic strategy. In addition to the overactivation of the endogenous antioxidant system, the effects demonstrated on body weight and blood glucose control reinforce the therapeutic potential of GT, considering that maintaining weight and blood glucose levels at physiological levels is a key component for the prevention and control of PN [3].

The present work was able to highlight the antioxidant effects of GT and its impact on the preservation of sensory fibers, a crucial aspect in the management of HFD-induced PN. The research uses a comprehensive approach, including behavioral, morphological, and molecular assessments, to further characterize the therapeutic effects of GT, which strengthens the credibility and depth of the findings. In addition, the study contributes significantly to the understanding of the mechanisms of action of GT, suggesting that its neuroprotective effects may be related to the regulation of endogenous antioxidant systems in nervous tissue. These results are consistent with the literature and pave the way for future research into GT’s bioactive compounds, such as catechins and caffeine, which appear to exert antinociceptive and neuroprotective effects. Although the study presents promising results, further studies on the mechanism of action of GT, investigating the signaling pathways involved, are still needed. In addition, the present study shows results from an experimental model in mice, so the therapeutic potential of GT for neuropathy still requires validation by clinical studies with high methodological quality. These findings demonstrate the strong potential of GT as a strategy for the control of sensory PN and associated metabolic markers and highlight the relevance of future clinical investigations.

## 5. Conclusions

Taken together, the data are consistent with the therapeutic effects of GT consumption in alleviating HFD-induced peripheral neuropathy. Daily administration of GT was able to induce an antinociceptive effect, control body weight and blood glucose levels, upregulate the endogenous antioxidant system in nervous tissue, and preserve the structure of sensory nerve fibers in neuropathic mice. These findings demonstrate the strong potential of GT as a strategy for the control of sensory PN and associated metabolic markers, and highlight the relevance of future clinical investigations.

## Data Availability

The raw data supporting the conclusions of this article will be made available by the authors on request.

## Supplementary Materials

The following supporting information can be downloaded at: https://www.mdpi.com/article/10.3390/antiox14040452/s1.

## Abbreviations

CAT	Catalase
CD	Control diet
CNS	Central nervous system
GLUT2	Glucose transporter 2
GPX	Glutathione peroxidase
GT	Green tea
HFD	High-fat diet
NRF-2	Nuclear factor erythroid 2-related factor 2
PYY	Peptide YY
PN	Peripheral neuropathy
RT-qPCR	Quantitative reverse transcription polymerase chain reaction
SD	Standard deviation
SGLT-1	Sodium-glucose transport proteins 1
SOD	Superoxide dismutase
