# Supplementary material for: Green Tea Intake Reduces High-Fat Diet-Induced Sensory Neuropathy in Mice by Upregulating the Antioxidant Defense System in the Spinal Cord
Source: Antioxidants (Basel). 2025 Apr 10;14(4):452. doi: 10.3390/antiox14040452 (PMC12023980; doi:10.3390/antiox14040452)
Supplement: Supplementary file 1 [file antioxidants-14-00452-s001.zip › antioxidants-3558853-supplementary.pdf]

Table S1. Composition of the control and high-fat diets.

High-fat diet			Control diet		
	g%	KCal		g%	KCal
Protein	23.4	17.5	Protein	16.8	16.4
Carbohydrate	33.2	24.69	Carbohydrate	74.3	73.1
Fat	34.6	57.9	Fat	4.8	10.5
Total (Kcal/g)	3.87	100	Total (Kcal/g)	4.07	100
